# The association of dietary approaches to stop hypertension (DASH) with the odds of diabetic nephropathy and metabolic markers in women: a case–control study

**DOI:** 10.1186/s12905-022-02140-y

**Published:** 2023-02-12

**Authors:** Atieh Mirzababaei, Faezeh Abaj, Sara Hajishizari, Niki Bahrampour, Sahar Noori, Ahmad Mujtaba Barekzai, Dorsa Hosseininasab, Cain C. T. Clark, Khadijeh Mirzaei

**Affiliations:** 1 Department of Community Nutrition, School of Nutritional Sciences and Dietetics, Tehran University of Medical Sciences (TUMS), P.O. Box 14155-6117, Tehran, Iran; 2 Department of Nutrition, Science and Research Branch, Islamic Azad University, Tehran, Iran; 3 Centre for Intelligent Healthcare, Coventry University, Coventry, CV1 5FB UK; 4 Food Microbiology Research Center, Tehran University of Medical Sciences (TUMS), Tehran, Iran

**Keywords:** Dietary approaches to stop hypertension, Diabetic nephropathy, Case–control

## Abstract

**Objectives:**

Epidemiologic studies have reported that diet is associated with diabetes and its complications through different pathways. We sought to examine the associations between the Dietary Approaches to Stop Hypertension (DASH) diet and the odds of diabetic nephropathy (DN) developing in Iranian women with existing type 2 diabetes.

**Methods:**

In this case–control study, 105 women with DN and 105 controls, matched for age and diabetes duration, were selected from the Kowsar Diabetes Clinic in Semnan, Iran. DASH, estimated using dietary intake, was assessed using a validated and reliable food frequency questionnaire with 147 items. Anthropometric measurements were assessed for all subjects. Logistic regression was performed to examine the association between DASH and the odds of developing DN.

**Results:**

After controlling for potential confounders, subjects in the highest intake of DASH diet adherence have 84% lower odds of DN, compared to those with the lowest intake (OR = 0.16, 95% CI = 0.07–0.34, *P* < 0.001). Among DASH diet subcategories, intakes of vegetables (80%), fruits (88%), nuts and legumes (87%), and low-fat dairy (73%) decreased the risk of DN after adjustment for confounders (*P* < 0.001).

**Conclusions:**

This study showed that the DASH diet is associated with lower odds of DN development in women with type 2 diabetes.

## Introduction

Diabetic nephropathy (DN) is one the most important of diabetes complications that can lead to renal dysfunction and end-stage renal disease (ESRD) in diabetic patients [1, 2]. In DN, proteins excrete from kidney [3], and high blood pressure (BP), decreasing kidney function, and excretion of more than 300 mg/day of protein are criteria for DN [2]. Many previous studies have indicated that family history, ethnicity, gestational diabetes, dyslipidemia, obesity, hypertension, and insulin resistance are major risk factors for DN [4]. About 50% of global ESRD is due to diabetes [5], and predictions indicate that 7.7% of the global population will have diabetes by 2030 [6–8]. Additional reports have demonstrated that the rate of diabetes will increase by about 70% between 2010 and 2030 [6–8], whilst according to a study in Iran, the prevalence of DN was reported to be about 31% [2]. DN, as one of the most serious microvascular complications of diabetes, accounts for 4 million mortalities per year in the world [8–10]. As the incidence of type 2 diabetes (T2D) increases, efforts to stop the progression of diabetes to diabetic kidney disease and ESRD are essential. Modification of risk factors, such as precise control of blood glucose, and management of hypertension and hyperlipidemia may contribute to delayed progression of diabetic kidney disease [6]. Diet can be used to control hypertension and blood glucose [11, 12], where it appears that greater adherence to plant-based diets and fewer processed foods can have positive affects diabetic nephropathy [13].

One of the diets that can help with DN is the dietary approaches to stop hypertension diet (DASH). In the DASH diet, individuals are encouraged to consume whole grains, fruits, vegetables, low-fat dairy products, legumes, seeds, fish, and poultry (lean meats), but, on the other hand, consumption of saturated fat, red meats, sweets, and sugar-sweetened beverages (SSBs) is limited [14, 15]. Higher amounts of protein, fiber, magnesium, calcium, potassium, antioxidant components, and unsaturated fatty acids in this diet, can help to reduce the risk of diabetes [16]. Studies have demonstrated that adherence to the DASH diet can prevent diabetes [11]. However, some clinical trials reported inconsistent results about the relationship between the DASH diet and diabetes [12, 17–19]. In one study, it was noted that adherence to the DASH diet, concomitant to an exercise program, can effect diabetes in persons with hypertension and overweight [20]. Further, blood pressure, as a risk factor for kidney diseases and diabetic nephropathy, is shown to be reduced by adherence to the DASH diet [21].

Given the high prevalence of diabetes and its progression to complications, such as DN and eventually ESRD, as well as the economic burden on health systems, it is important to discern preventative measures for DN progression. Since the DASH diet is effective in controlling both diabetes and BP, which are serious factors in the progression of DN, we sought to examine the relationship between DASH diet and odds of DN.

## Methods

### Study population

In the current case–control study, 210 women (105 cases and 105 controls) were recruited from the Kowsar Diabetes Clinic by convenience sampling in Semnan, Iran. 105 diabetic women without DN were recruited as the control group by a 1:1 matching to the DN cases, by age at 1-year intervals and by the duration of diabetes in 6-months intervals, from the same center. The inclusion criteria were women with T2D, aged between 30 and 65 years, and with a history of 3–10 years of T2D. The definition of diabetes used in this study is based on the American Diabetes Association (ADA) criteria: 2-h post-load blood glucose (2hrBG) ≥ 200 mg/dl or fasting blood glucose (FBG) ≥ 126 mg/dl; glycosylated hemoglobin (HbA1c) ≥ 6.5% [22]. The exclusion criteria were having autoimmune disorders or previous history of cancer, coronary angiography, hepatic disease, myocardial infarction, or stroke. Total energy intake of < 500 or > 3500 kcal/day and/or poor response to the food-frequency questionnaire (FFQ) were also considered as exclusion criteria. DN was defined as urinary albumin-to-creatinine ratio (ACR) ≥ 30 mg/g in a random spot urine sample in the present study [23]. This work was approved by the Ethics Committee of Tehran University of Medical Sciences (Ethics Number: IR.TUMS.REC.1395.2644) and the Ethics Committee of Semnan University of Medical Sciences (Ethics Number: IR. SEMUMS. REC.1395.66) and was conducted in line with the guidelines of the Declaration of Helsinki.

### Dietary intake assessment and DASH diet score calculation

A validated FFQ was used to assess dietary intake of participants [24]. Then, participants reported their intake of food items daily, weekly, monthly, or yearly. Portion sizes were finally converted into g/day using household measurements. After that, these amounts were adjusted for energy intake using the residual method [25]. For estimating the energy and nutrient intakes, dietary intakes were analyzed using NUTRITIONIST 4 (First Data Bank, San Bruno, CA) software. For computing the DASH diet score, the components were classified into groups, based on their intake ranking. The component score for nuts and legumes, fruits, vegetables, low-fat dairy products, and whole grains were ranked by decile. For example, decile 1 was assigned 1 point and decile 10, 10 points. For sodium, sweetened beverages, red and processed meats, lower intakes were desired. Hence, the lowest decile was given a score of 10 points and the highest 0 points. The component scores were finally summed to obtain an overall DASH score, ranging from 0 to 80 [26].

### Assessment of other variables

Age, diabetes duration, medical history, and current drug usage were recorded by trained interviewers. Weight (kg) was measured while subjects were wearing light clothing, without shoes. Body mass index (BMI, kg/m^2^) was computed as weight (kg) divided by the square of height (m). After a resting period ≥ 5 min, BP was measured on the left arm using a manual sphygmomanometer. A validated physical activity questionnaire (IPAQ) [27] was utilized to evaluate individuals’ physical activity (PA).

### Blood biomarkers assessment

Participant’s past 3 months medical records were used to obtain their fasting blood sugar (FBS), 2hrBG, HbA1c, total cholesterol (TC), triglycerides (TG), low-density lipoprotein (LDL), high-density lipoprotein (HDL), total serum creatinine (Cr), and blood urea nitrogen (BUN) levels.

### Statistical analysis

The distribution of the quantitative variables was assessed using the Kolmogorov–Smirnov test. Independent samples T tests and chi-square tests were used to compare quantitative and qualitative variables between cases and controls, and they were presented as mean ± SD and frequency (%), respectively. Energy-adjusted dietary intakes, across DASH diet scores, were compared using independent samples T tests. To investigate the relationship between DASH diet and DN, logistic regression was used to determine the odds ratio (OR) of DN and its 95% confidence interval (CI).

Linear regression analysis was used to determine the relationship between DASH score and its subcategories with biochemical markers, in crude and adjusted models. In adjusted models, energy intake, age, PA, BMI, cardiovascular disease history, and type of drug used (angiotensin receptor blockers, angiotensin-converting enzyme inhibitors, beta-blockers, metformin, sulphonylurea, and insulin) were controlled. Data analysis was performed using SPSS software (Version 25, SPSS Inc., Chicago, IL, USA) and *P* < 0.05 was, a priori, considered statistically significant.

## Results

### Participants and study characteristics across case and control group

**Fig. 1:**
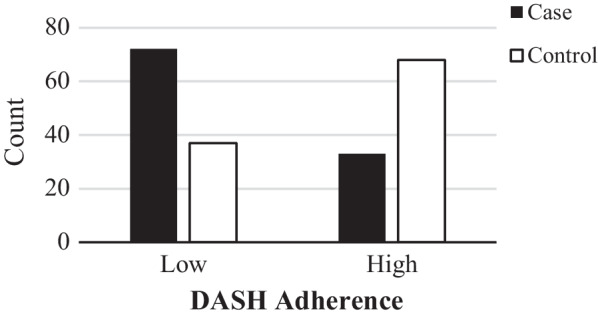
The frequency of cases and controls across lower and higher adherence of DASH diet

Overall, 210 subjects were enrolled in the study, including 105 cases and 105 control with DM. The basic characteristics of the participants, according to case and control status, are presented in Table 1. There was a significant difference between serum albumin, ACR, Hb1Ac, LDL, creatinine, Angiotensin receptor blockers (ARBs), and Angiotensin-converting enzyme (ACEIs) usage (*P* < 0.001). The frequency of cases and controls across following DASH diet are shown in Fig. 1. Counts of patients above vs below the median of DASH diet scores were significantly different (*P* < 0.001). 64.5% of subjects who were in the lower adherence group had DN, whilst 35.5% of patients with higher adherence had DN. On other hand, mean ± SD of age, BMI, and diabetes duration of cases was 55.33 ± 7.04 (years), 28.686 ± 4.74 (kg/m^2^), and 7.60 ± 2.21 (years), respectively (*P* > 0.05). Finally, mean ± SD of FBS (167.10 ± 50.62) and cholesterol level (185.15 ± 38.12) (mg/dl) were marginally higher among the cases group (*P* < 0.05).

**Table 1:** General characteristics of population based on case and control groups

	Control (n = 105)	Case (n = 105)	*P* value
*Demographic characteristic*			
Age (y)	55.41 ± 7.14	55.33 ± 7.04	0.94
Albumin (g/dl)	8.37 ± 6.76	14.40 ± 11.94	< **0.001**
ACR	18.66 ± 5.92	232.18 ± 114.07	< **0.001**
Diabetes duration (y)	7.56 ± 2.17	7.60 ± 2.21	0.88
*Blood pressure*			
SBP (mmHg)	129.04 ± 98.88	126.59 ± 17.27	0.80
DBP (mmHg)	80.10 ± 11.76	82.80 ± 13.09	0.12
*Anthropometry characteristic*			
Body weight (kg)	71.589 ± 11.50	73.400 ± 13.83	0.30
Height (cm)	161.17 ± 5.91	160.68 ± 6.29	0.56
BMI (kg/m^2^)	27.510 ± 4.39	28.686 ± 4.74	0.06
*Blood parameters*			
Hb (mg/dl)	12.630 ± 1.22	12.610 ± 1.37	0.91
FBS (mg/dl)	154.19 ± 45.03	167.10 ± 50.62	**0.05**
BS (mg/dl)	207.10 ± 54.35	217.75 ± 53.23	0.15
HbA1c (%)	8.031 ± 1.29	8.660 ± 1.41	< **0.001**
Cholesterol (mg/dl)	175.38 ± 32.42	185.15 ± 38.12	**0.05**
TG (mg/dl)	162.25 ± 57.91	167.26 ± 65.68	0.56
LDL (mg/dl)	94.60 ± 29.47	106.86 ± 31.77	< **0.001**
HDL (mg/dl)	46.37 ± 9.25	45.05 ± 9.26	0.30
Cr (mg/dl)	0.87 ± 0.17	0.92 ± 0.16	**0.03**
BUN (mg/dl)	15.17 ± 3.86	15.79 ± 4.55	0.29
*Qualitative variables*			
PA (met-h/w)			
Low (> 600)	37 (17.6)	31 (14.8)	0.12
Moderate (600–3000)	28 (13.4)	42 (20)	
High (< 3000)	40 (19)	32 (15.2)	
*Medical history*			
Positive CVD history	23 (11)	24 (11.4)	0.86
*Medication usage*			
ARBs	45 (21.4)	60 (28.6)	**0.03**
ACEIs	21 (10)	44 (21)	**0.001**
Beta blockers	18 (8.6)	20 (9.5)	0.56
Metformin	104 (49.5)	104 (49.5)	0.75
sulfonylurea	62 (29.5)	71 (33.8)	0.19
Insulin	35 (16.7)	26 (12.4)	0.17

Data are presented as mean ± SD or number (percent)

Independent sample T test and chi square were used

Significant items with a* P* value ≤ 0.05 are bolded

*ACR* Albumin creatinine ratio, *SBP* Systolic blood pressure, *DBP* Diastolic blood pressure, *BMI* Body mass index, *Hb* Hemoglobin, *FBS* Fasting blood sugar, *BS* Blood sugar, *TG* Triglycerides, *HDL* High density lipoprotein, *LDL* Low density lipoprotein, *CR* Creatinine, *BUN* Blood urea nitrogen, *DASH* Dietary approach to stop hypertension, *PA* Physical activity, *met* Metabolic equivalent, *CVD* Cardiovascular disease, *ARBs* Angiotensin receptor blockers, *ACEIs* Angiotensin converting enzyme inhibitors

### Dietary intakes of cases and controls across lower and higher adherence of DASH diet

The DASH diet subgroups, macronutrients, fat and water soluble vitamins, minerals, and other parts of dietary intakes were reported in Table 2. Overall, the adherence to DASH diet was divided to two groups of above and below the median. Among DASH diet subcategories, consumption of fruits, vegetables, nuts and legumes, and dairy products were increased in both case and control groups (*P* < 0.05). Meat intake was decreased among case and control groups, whilst sweetened beverages and whole grains intake were significantly different between lower and higher adherence of DASH diet in the case group with DN (*P* < 0.05).

**Table 2:** Dietary intakes of cases and controls across low and high adherence of DASH diet

	Control			Case		
	Low (n = 37)	High (n = 68)	*P* value	Low (n = 72)	High (n = 33)	*P* value
*DASH diet subcategories*						
Fruits (g/d)	4.67 ± 1.97	7.47 ± 2.20	< **0.001**	3.09 ± 2.07	6.36 ± 2.46	< **0.001**
Vegetables (g/d)	266.95 ± 78.65	447.66 ± 143.61	< **0.001**	208.07 ± 78.91	440.27 ± 321.26	< **0.001**
Nuts and legumes (g/d)	125.53 ± 60.17	164.11 ± 77.17	**0.01**	87.81 ± 35.71	112.69 ± 57.53	**0.01**
Low-fat dairy (g/d)	185.28 ± 63.53	350.18 ± 138.59	< **0.001**	145.83 ± 101.80	314.54 ± 134.49	< **0.001**
Whole grains (g/d)	46.90 ± 66.98	81.02 ± 111.26	0.09	22.14 ± 33.68	115.39 ± 114.67	< **0.001**
Sodium (mg/d)	4.18 ± 1.62	3.19 ± 3.57	0.11	4.25 ± 2.65	3.66 ± 1.88	0.26
Red meat (g/d)	30.47 ± 11.28	21.79 ± 20.08	**0.01**	38.00 ± 8.91	21.30 ± 13.85	< **0.001**
Sweetened beverages (g/d)	146.13 ± 386.34	121.06 ± 267.43	0.69	38.97 ± 31.13	21.28 ± 36.12	**0.01**
*Dietary intakes*						
Energy (kcal)	1609.46 ± 254.47	1370.40 ± 325.37	< **0.001**	1423.22 ± 235.42	1364.38 ± 287.03	0.27
*Macronutrients*						
Protein (g)	50.93 ± 7.04	46.83 ± 11.17	**0.04**	45.34 ± 6.63	46.73 ± 10.57	0.42
Carbohydrate (g)	286.61 ± 58.54	241.15 ± 66.74	**0.001**	250.32 ± 37.58	236.95 ± 48.58	0.13
Fat (g)	35.39 ± 6.73	31.00 ± 6.47	**0.002**	33.30 ± 9.29	32.34 ± 8.74	0.62
*Fat subcategories*						
Cholesterol (g)	8.70 ± 8.79	8.12 ± 11.30	0.79	5.32 ± 3.56	5.02 ± 6.71	0.77
SFA (g)	6.76 ± 1.30	5.95 ± 1.56	**0.009**	6.22 ± 1.72	5.75 ± 1.84	0.21
MUFA (g)	11.84 ± 2.69	10.41 ± 2.63	**0.01**	11.31 ± 3.68	10.49 ± 3.06	0.27
PUFA (g)	10.70 ± 2.12	10.19 ± 1.87	0.20	10.32 ± 1.90	10.89 ± 3.78	0.32
Oleic acid (g)	11.50 ± 2.62	10.00 ± 2.38	**0.004**	11.01 ± 3.55	10.30 ± 3.04	0.32
Linoleic acid (g)	9.62 ± 1.83	9.07 ± 1.58	0.11	9.22 ± 1.67	9.87 ± 3.71	0.23
Linolenic acid (g)	0.91 ± 0.28	0.92 ± 0.32	0.84	0.92 ± 0.24	0.87 ± 0.32	0.47
*Minerals*						
Sodium (mg)	3991.56 ± 691.60	3081.65 ± 925.41	< **0.001**	3764.85 ± 1136.80	3480.92 ± 883.52	0.21
Potassium (mg)	1716.65 ± 418.61	1725.28 ± 449.35	0.92	1652.28 ± 257.03	1729.52 ± 467.58	0.29
Calcium (mg)	386.98 ± 77.75	448.01 ± 62.49	< **0.001**	377.62 ± 76.05	408.14 ± 62.78	**0.03**
Iron (mg)	16.05 ± 2.17	14.47 ± 2.75	**0.004**	14.78 ± 1.89	14.87 ± 2.55	0.85
Phosphorus (mg)	953.96 ± 137.22	890.58 ± 189.50	0.07	899.73 ± 121.97	920.51 ± 201.27	0.52
Magnesium (mg)	359.20 ± 67.66	349.44 ± 69.84	0.49	345.09 ± 48.83	378.49 ± 121.04	**0.05**
Zinc (mg)	8.36 ± 1.42	8.07 ± 1.61	0.36	7.70 ± 1.18	9.01 ± 4.51	**0.02**
Copper (mg)	1.61 ± 0.26	1.51 ± 0.25	**0.05**	1.51 ± 0.22	1.60 ± 0.32	0.10
Selenium (microgram)	130.95 ± 20.22	116.64 ± 34.76	**0.02**	122.87 ± 19.97	124.38 ± 30.70	0.76
*Water soluble vitamins*						
C (mg)	9.22 ± 6.48	14.53 ± 7.11	< **0.001**	8.30 ± 3.17	10.43 ± 4.30	**0.01**
B1 (mg)	1.89 ± 0.31	1.62 ± 0.39	< **0.001**	1.68 ± 0.25	1.65 ± 0.32	0.69
B2 (mg)	1.09 ± 0.18	0.92 ± 0.21	< **0.001**	0.97 ± 0.17	0.95 ± 0.16	0.58
B3 (mg)	17.82 ± 2.57	15.19 ± 3.38	< **0.001**	16.19 ± 2.57	15.93 ± 2.90	0.65
B5 (mg)	2.61 ± 0.53	2.42 ± 0.68	0.14	2.39 ± 0.67	2.64 ± 0.71	0.08
B6 (mg)	0.78 ± 0.18	0.74 ± 0.12	0.12	0.78 ± 0.12	0.79 ± 0.16	0.75
B9 (microgram)	380.15 ± 111.88	445.12 ± 115.97	**0.006**	349.61 ± 62.77	371.32 ± 66.59	0.12
B12 (microgram)	0.19 ± 0.15	0.17 ± 0.20	0.63	0.13 ± 0.07	0.10 ± 0.12	0.17
*Fat soluble vitamins*						
A (RAE)	20.02 ± 18.58	24.12 ± 11.62	0.17	15.70 ± 11.38	24.76 ± 11.85	< **0.001**
E (mg)	4.48 ± 1.98	4.10 ± 1.80	0.32	3.91 ± 0.91	4.00 ± 1.09	0.66
K (microgram)	13.03 ± 5.04	13.52 ± 4.67	0.62	11.49 ± 2.98	13.73 ± 6.01	**0.04**
*Simple carbohydrates*						
Fructose (g)	16.20 ± 19.62	14.52 ± 13.56	0.61	11.85 ± 2.71	10.30 ± 2.60	**0.008**
Sucrose (g)	15.22 ± 8.96	9.68 ± 5.47	< **0.001**	16.00 ± 7.12	11.08 ± 6.68	**0.001**
Glucose (g)	14.81 ± 18.64	13.37 ± 12.69	0.64	10.78 ± 2.64	9.14 ± 2.48	**0.004**
Total fiber (g)	41.81 ± 11.97	37.80 ± 8.65	**0.05**	38.39 ± 6.87	36.20 ± 7.63	0.15

Data are presented as mean ± SD. Low and high adherence of median of DASH diet are shown

Independent T test was used

Significant items with a* P* value ≤ 0.05 are bolded

*SFA* Saturated fatty acids, *PUFA* Poly unsaturated fatty acids, *MUFA* Monounsaturated fatty acids

The DN cases consumed significantly more magnesium and zinc (*P* < 0.05). In addition, energy, protein, carbohydrate, and fat intake were decreased with increasing DASH diet adherence in the control group (*P* < 0.05). Among simple sugars, consumption of sucrose was significantly reduced in higher DASH diet adherence in both cases and controls (*P* < 0.05).

### Baseline characteristics of cases and controls groups among lower and higher adherence of median of DASH diet

The general characteristics and dietary intakes of the participants in increasing adherence of the DASH diet were shown in Table 3. The participants in the highest column of the DASH diet had lower albumin serum level (6.11 ± 5.097), height (159.93 ± 6.177), BUN (0.87 ± 0.18), and higher creatinine level (0.87 ± 0.18), after adjusting for confounders including age, energy intake, and physical activity, across the control group (*P* < 0.05). Compared to the control group, diastolic blood pressure (DBP), FBS, and HbA1c levels were decreased in case groups after adjusting for mentioned confounders, aligned with higher adherence to the DASH diet(*P* < 0.05).

**Table 3:** Baseline characteristics of cases and controls groups among low and high adherence of DASH diet

	Control				Case			
	Low (n = 37)	High (n = 68)	*P* value	*P* value*	Low (n = 72)	High (n = 33)	*P* value	*P* value*
*Demographic characteristic*								
Age (y)	54.06 ± 6.555	56.12 ± 7.428	0.16	–	55.44 ± 7.082	54.76 ± 7.483	0.65	–
Albumin (g/dl)	12.58 ± 7.602	6.11 ± 5.097	< **0.001**	< **0.001**	15.56 ± 13.158	13.09 ± 10.001	0.34	0.34
ACR	19.65 ± 6.212	18.09 ± 5.766	0.20	0.07	239.74 ± 91.266	217.67 ± 145.891	0.35	0.42
Diabetes duration (y)	7.67 ± 2.111	7.49 ± 2.222	0.70		7.65 ± 2.184	7.50 ± 2.305	0.75	
*Blood pressure*								
SBP (mmHg)	121.72 ± 16.554	133.00 ± 122.428	0.58	0.69	128.61 ± 16.597	123.64 ± 19.272	0.18	0.08
DBP (mmHg)	81.53 ± 13.064	79.25 ± 11.096	0.35	0.67	84.83 ± 14.064	78.33 ± 9.746	**0.01**	**0.04**
*Anthropometry characteristic*								
Body weight (kg)	69.686 ± 12.9626	72.722 ± 10.6348	0.20	0.30	73.561 ± 12.4596	72.182 ± 16.2292	0.64	0.63
Height (cm)	163.36 ± 4.673	159.93 ± 6.177	**0.004**	< **0.001**	160.35 ± 6.348	161.79 ± 6.294	0.28	0.36
BMI (kg/m^2^)	26.028 ± 4.9568	28.367 ± 3.8442	**0.009**	0.07	28.586 ± 4.7848	28.356 ± 3.9868	0.81	0.79
*Blood parameters*								
Hb (mg/dl)	12.456 ± 1.2796	12.753 ± 1.1719	0.23	0.19	12.439 ± 1.3583	13.112 ± 1.3188	**0.02**	0.18
FBS (mg/dl)	161.92 ± 45.779	149.81 ± 44.668	0.19	0.57	168.71 ± 53.292	160.79 ± 47.320	0.47	**0.03**
BS (mg/dl)	217.22 ± 52.193	201.62 ± 55.460	0.16	0.36	220.26 ± 58.710	210.42 ± 43.663	0.39	0.07
HbA1c (%)	8.44 ± 1.21	7.79 ± 1.28	**0.01**	0.10	8.74 ± 1.38	8.33 ± 1.44	0.17	**0.02**
Cholesterol (mg/dl)	180.69 ± 25.829	172.26 ± 35.373	0.21	0.39	187.83 ± 36.408	183.45 ± 41.200	0.59	0.87
TG (mg/dl)	164.31 ± 49.132	159.75 ± 61.630	0.70	0.87	163.83 ± 56.674	171.91 ± 81.388	0.56	0.41
LDL (mg/dl)	103.97 ± 29.577	89.10 ± 28.167	**0.01**	0.10	108.80 ± 33.345	103.73 ± 27.924	0.45	0.91
HDL (mg/dl)	46.00 ± 8.903	46.59 ± 9.553	0.76	0.93	44.88 ± 9.421	43.82 ± 8.041	0.58	0.87
Cr (mg/dl)	0.86 ± 0.14	0.87 ± 0.18	0.79	**0.03**	0.9480 ± 0.16718	0.8679 ± 0.14339	**0.02**	0.18
BUN (mg/dl)	16.50 ± 4.398	14.43 ± 3.374	**0.009**	**0.007**	15.67 ± 4.086	15.76 ± 5.657	0.93	0.89
*Qualitative variables*								
PA (met-h/w)								
Low (> 600)	13 (35.1)	24 (64.9)	0.79		20 (66.7)	10 (33.3)	0.89	
Moderate (600–3000)	9 (29.6)	19 (70.4)			33 (67.5)	13 (32.5)		
High (< 3000)	15 (37.5)	25 (62.5)			19 (66.5)	10 (34.5)		
*Medical history*								
Positive CVD history	10 (43.5)	13 (56.5)	0.31		14 (63.6)	8 (36.4)	0.73	
*Medication usage*								
ARBs	16 (35.6)	29 (64.4)	0.86		36 (64.3)	20 (35.7)	0.56	
ACEIs	9 (45)	11 (55)	0.27		29 (67.4)	14 (32.6)	0.88	
Beta blockers	7 (38.9)	11 (61.1)	0.67		13 (68.4)	6 (31.6)	0.36	
Metformin	35 (34)	68 (66)	0.34		65 (66.3)	33 (33.7)	0.99	
sulfonylurea	22 (36.1)	39 (63.9)	0.71		43 (65.2)	23 (34.8)	0.65	
Insulin	12 (34.3)	23 (65.7)	0.96		20 (76.9)	6 (23.1)	0.19	

Data are presented as mean ± SD or number (percent). Low and high adherence of median of DASH diet are shown

Independent sample T test and chi square were used

Significant items with a* P* value ≤ 0.05 are bolded

*ACR* Albumin creatinine ratio, *SBP* Systolic blood pressure, *DBP* Diastolic blood pressure, *BMI* Body mass index, *Hb* Hemoglobin, *FBS* Fasting blood sugar, *BS* Blood sugar, *TG* Triglycerides, *HDL* High density lipoprotein, *LDL* Low density lipoprotein, *CR* Creatinine, *BUN* Blood urea nitrogen, *CVD* Cardiovascular disease, *ARBs* Angiotensin receptor blockers, *ACEIs* Angiotensin converting enzyme inhibitors

*Adjusted for age, energy intake, physical activity

### Association between adherence to a DASH-style diet and the odds of DN

The ORs for DN across the lowest and highest median of the DASH diet subcategories were detailed in Table 4. In the crude model of total DASH diet, participants in the high adherence group of the DASH diet were 74% less likely to have DN (ORs: 0.26; 95% CI 0.14–0.47; *P* < 0.001). After controlling for energy intake, age, physical activity, and BMI, adherence to the DASH diet remained negatively associated with the odds of DN (ORs: 0.20; 95% CI 0.10–0.38; *P* < 0.001) (Model 1). In model 2, which was adjusted for model 1 + diabetes duration, cardiovascular diseases history, and drug usage (ARBs, ACEIs, beta-blockers, metformin, sulphonyl urea, and insulin), the inverse association remained (OR: 0.16; 95% CI 0.07–0.34; *P* < 0.001). Among DASH diet subcategories, intakes of vegetables (80%), fruits (88%), nuts and legumes (87%), and low-fat dairy (73%) decreased the risk of DN in model 2 (*P* < 0.001). In addition, intake of whole grains was inversely associated with high risk of DN among women (OR: 0.51; 95% CI 0.27–0.95; *P* = 0.03) in model 2. Moreover, logistic regression showed that red meat consumption increased the risk of DN in the model 2 (OR: 1.20; 95% CI 0.10–0.41; *P* < 0.001).

**Table 4:** Odds ratio and 95% confidence intervals of diabetic nephropathy according to following DASH diet among subjects

DASH diet			
Variables	Low	High OR (95% CI)	*P* value
N (case/control)	(72/37)	(33/68)	–
*DASH score*			
Crude model	1.00 (Ref)	0.26 (0.14–0.47)	< **0.001**
Model 1	1.00 (Ref)	0.20 (0.10–0.38)	< **0.001**
Model 2	1.00 (Ref)	0.16 (0.07–0.34)	< **0.001**
*Vegetables (g/d)*			
Crude model	1.00 (Ref)	0.27 (0.15–0.48)	< **0.001**
Model 1	1.00 (Ref)	0.24 (0.13–0.43)	< **0.001**
Model 2	1.00 (Ref)	0.20 (0.10–0.39)	< **0.001**
*Fruits (g/d)*			
Crude model	1.00 (Ref)	0.23 (0.13–0.42)	< **0.001**
Model 1	1.00 (Ref)	0.17 (0.08–0.32)	< **0.001**
Model 2	1.00 (Ref)	0.12 (0.05–0.26)	< **0.001**
*Nuts and legumes (g/d)*			
Crude model	1.00 (Ref)	0.17 (0.09–0.32)	< **0.001**
Model 1	1.00 (Ref)	0.15 (0.08–0.30)	< **0.001**
Model 2	1.00 (Ref)	0.13 (0.06–0.28)	< **0.001**
*Low-fat dairy*			
Crude model	1.00 (Ref)	0.36 (0.20–0.63)	< **0.001**
Model 1	1.00 (Ref)	0.33 (0.18–0.60)	< **0.001**
Model 2	1.00 (Ref)	0.27 (0.14–0.52)	< **0.001**
*Whole grains (g/d)*			
Crude model	1.00 (Ref)	0.55 (0.31–0.96)	**0.03**
Model 1	1.00 (Ref)	0.58 (0.32–1.14)	**0.06**
Model 2	1.00 (Ref)	0.51 (0.27–0.95)	**0.03**
*Sodium (mg/d)*			
Crude model	1.00 (Ref)	0.61 (0.32–1.17)	0.14
Model 1	1.00 (Ref)	0.63 (0.32–1.22)	0.17
Model 2	1.00 (Ref)	0.77 (0.37–1.57)	0.47
*Red meat (g/d)*			
Crude model	1.00 (Ref)	1.31 (0.17–0.55)	0.12
Model 1	1.00 (Ref)	1.27 (0.15–0.50)	< **0.001**
Model 2	1.00 (Ref)	1.20 (0.10–0.41)	< **0.001**
*Sweetened beverages*			
Crude model	1.00 (Ref)	1.12 (0.65–1.94)	0.67
Model 1	1.00 (Ref)	1.02 (0.57–1.82)	0.92
Model 2	1.00 (Ref)	1.15 (0.62–2.15)	0.64

Logistic regression was used. Significant items with a* P* value ≤ 0.05 are bolded

Data are presented as odds ratio (95% confidence interval)

Lower and higher adherence of median of DASH diet are shown

Model 1: Adjusted for energy intake, age, physical activity, body mass index

Model 2: model 1 + diabetes duration, cardiovascular diseases history, and drug usage (angiotensin receptor blockers; angiotensin converting enzyme inhibitors, beta-blockers, metformin, sulphonyl urea, and insulin)

### Association between DASH-style diet and biochemical markers of participants

The association between DASH score and subcategories with biochemical markers, are presented in Table 5. There was no significant association between FBS, TG, HDL, total cholesterol and BUN with DASH score and subcategories (*P* > 0.05). However, there was an inverse relationship between DASH score and both creatinine (β = − 0.08, 95% CI  = − 0.14, − 0.01, *P* value = 0.02) and DBP (β = − 6.50, 95% CI  = − 11.91, − 1.08, *P* value = 0.01), after adjusting for potential confounders the results remained significant. Among DASH diet subcategories, low fat dairy was inversely related to LDL (β = − 16.28, 95% CI  = − 29.7, − 2.77, *P* value = 0.01) (in model 2), DBP (β = − 6.41, 95% CI  = − 11.63, − 1.20, *P* value = 0.01), and Alb (β = − 6.85, 95% CI  = − 2.11, − 11.59, *P* value = 0.005) (in crude and adjusted model). Additionally, vegetables maintained negative association with ACR (β = − 45.05, 95% CI  = − 90.95, 0.84, *P* value = 0.04), and SBP (β = − 9.09, 95% CI  = − 15.94, − 2.25, *P* value = 0.01) after adjusting in model 1 and 2. Fruits also displayed a negative association with creatinine in model 2 (β = − 0.06, 95% CI  = − 0.13, − 0.001, *P* value = 0.04). Moreover, nuts and legumes were inversely associated with ACR (β = − 76.73, 95% CI  = − 130.05, − 23.42, *P* value = 0.005) and Alb (β = − 7.04, 95% CI  = − 12.82, − 1.27, *P* value = 0.01), after adjusting for confounders, the results remained significant. Whole grains had a negative relationship with both creatinine after adjustment (β = − 0.07, 95% CI  = − 0.14, 0.01, *P* value = 0.02), and DBP (β = − 6.47, 95% CI  = − 11.72, − 1.22, *P* value = 0.01). Besides, sodium demonstrated a positive association with DBP (β = − 6.58, 95% CI  = − 13.19, − 0.51, *P* value = 0.03), which remained significant after adjusting. Red meat was also positively related to creatinine (β = − 0.096, 95% CI  = − 0.16, 0.02, *P* value = 0.009) after adjusting in model 2.

**Table 5:** Association between DASH-style diet and biochemical markers of participants

Variables	DASH score			Vegetables			Fruits			Nuts and Legumes			Low fat dairy		
	β	95% CI	*P* value	β	95% CI	*P* value	β	95% CI	*P* value	β	95% CI	*P* value	β	95% CI	*P* value
*FBS*															
Crude	− 7.92	− 29.67 to 13.82	0.47	− 10.68	− 31.13 to 9.95	0.30	− 6.67	− 27.67 to 14.31	0.53	− 12.73	− 37.89 to 12.42	0.31	− 5.31	− 26.03 to 15.39	0.61
Model 1	− 3.82	− 25.08 to 17.43	0.72	− 11.53	− 31.32 to 8.24	0.25	− 7.28	− 27.51 to 12.93	0.47	− 14.84	− 38.96 to 9.28	0.22	− 8.22	− 28.25 to 11.80	0.41
Model 2	0.54	− 19.82 to 20.92	0.95	− 9.52	− 28.49 to 9.43	0.32	− 5.98	− 25.78 to 13.82	0.55	− 17.21	− 40.84 to 6.43	0.15	− 1.83	− 22.84 to 19.17	0.86
*TG*															
Crude	8.07	− 19.79 to 35.94	0.56	− 1.78	− 28.69 to 25.12	0.89	8.71	− 18.53 to 35.95	0.52	8.82	− 23.33 to 40.98	0.58	− 17.13	− 43.83 to 9.57	0.20
Model 1	7.82	− 20.28 to 35.94	0.58	− 1.81	− 28.61 to 24.99	0.89	9.47	− 17.74 to 36.69	0.49	11.28	− 20.97 to 43.53	0.48	− 15.54	− 42.40 to 11.32	0.25
Model 2	9.18	− 20.54 to 38.91	0.54	− 1.16	− 29.01 to 26.66	0.93	11.06	− 17.04 to 39.16	0.43	8.19	− 25.32 to 41.72	0.62	− 15.11	− 44.80 to 14.58	0.31
*HDL*															
Crude	− 1.06	− 4.86 to 2.74	0.58	0.09	− 3.69 to 3.89	0.96	− 0.50	− 4.35 to 3.34	0.79	2.32	− 2.18 to 6.84	0.30	− 1.34	− 5.12 to 2.44	0.48
Model 1	− 1.05	− 4.94 to 2.84	0.59	0.05	− 3.79 to 3.90	0.97	− 0.62	− 4.54 to 3.28	0.75	2.08	− 2.50 to 6.68	0.36	− 1.48	− 5.36 to 2.38	0.44
Model 2	− 0.49	− 4.18 to 3.19	0.79	0.20	− 3.48 to 3.89	0.91	− 0.53	− 4.27 to 3.19	0.77	2.72	− 1.56 to 7.01	0.21	− 0.34	− 4.29 to 3.61	0.86
*LDL*															
Crude	− 5.07	− 18.47 to 8.32	0.45	− 7.22	− 20.16 to 5.71	0.27	− 6.009	− 19.16 to 7.14	0.36	4.31	− 11.29 to 19.93	0.58	− 7.77	− 20.70 to − 5.15	0.23
Model 1	− 5.14	− 18.86 to 8.57	0.45	− 7.39	− 20.51 to 5.72	0.26	− 5.50	− 18.89 to 7.88	0.41	4.42	− 11.45 to 20.30	0.58	− 7.61	− 20.84 to − 5.61	0.25
Model 2	− 5.41	− 18.69 to 7.87	0.42	0.28	− 19.83 to 5.81	0.28	− 4.54	− 17.65 to 8.56	0.49	6.47	− 8.88 to 21.83	0.40	− 16.28	− 29.7 to − 2.77	**0.01**
*Total cholesterol*															
Crude	− 4.37	− 20.48 to 11.72	0.59	− 6.65	− 22.23 to 8.90	0.39	− 10.31	− 26.03 to 5.39	0.19	6.04	− 12.53 to 24.63	0.52	− 3.82	− 19.42 to 11.77	0.62
Model 1	− 4.12	− 20.60 to 12.36	0.62	− 6.42	− 22.29 to 9.36	0.42	− 10.27	− 26.30 to 5.76	0.20	6.64	− 12.29 to 25.58	0.48	− 4.04	− 20.04 to 11.59	0.61
Model 2	− 6.58	− 22.49 to 9.32	0.41	− 7.74	− 23.07 to 7.57	0.31	− 10.13	− 25.85 to 5.59	0.20	7.78	− 10.69 to 26.17	0.40	− 11.54	− 28.17 to 5.07	0.17
*HbA1C*															
Crude	− 0.41	− 1.002 − .18	0.17	− 0.54	− 1.11 to 0.02	**0.06**	− 0.18	− 0.77 to 0.39	0.52	− 0.20	− 0.90 to 0.50	0.57	− 0.20	− 0.77 to 0.37	0.49
Model 1	− 0.30	− 0.89 to 0.27	0.29	− 0.56	− 1.11 to − 0.02	**0.04**	− 0.19	− 0.75 to 0.36	0.49	− 0.25	− 0.92 to 0.41	0.44	− 0.29	− 0.84 to 0.26	0.30
Model 2	− 0.31	− 0.90 to 0.27	0.28	− 0.55	− 1.09 to − 0.004	**0.04**	− 0.18	− 0.75 to 0.37	0.51	− 0.20	− 0.88 to 0.48	0.55	− 0.32	− 0.92 to 0.27	0.28
*Creatinine*															
Crude	− 0.08	− 0.14 to 0.01	**0.02**	− 0.02	− 0.08 to 0.04	0.51	− 0.06	− 0.12 to 0.004	0.06	− 0.03	− 0.11 to 0.04	0.43	− 0.01	− 0.08 to 0.05	0.63
Model 1	− 0.07	− 0.14 to − 0.007	**0.03**	− 0.02	− 0.09 to 0.04	0.50	− 0.06	− 0.13 to 0.005	0.06	− 0.03	− 0.11 to 0.04	0.40	− 0.01	− 0.08 to 0.05	0.59
Model 2	− 0.08	− 0.15 to − 0.01	**0.01**	− 0.02	− 0.09 to 0.04	0.45	− 0.06	− 0.13 to − 0.001	**0.04**	− 0.03	− 0.12 to 0.04	0.37	− 0.04	− 0.11 to 0.02	0.17
*BUN*															
Crude	0.08	− 1.88 to 2.05	0.93	− 0.95	− 2.80 to 0.90	0.31	− 0.52	− 2.41 to 1.35	0.57	− 0.91	− 3.16 to 1.34	0.42	1.28	− 0.55 to 3.13	0.17
Model 1	0.20	− 1.81 to 2.22	0.83	− 0.97	− 2.85 to 0.91	0.30	− 0.56	− 2.49 to 1.36	0.56	− 0.93	− 3.23 to 1.37	0.42	1.29	− 0.60 to 3.19	0.17
Model 2	− 0.11	− 2.08 to 1.85	0.91	− 1.06	− 2.89 to 0.75	0.24	− 0.69	− 2.55 to 1.16	0.45	− 0.66	− 2.88 to 1.55	0.55	0.60	− 1.36 to 2.57	0.54
*ACR*															
Crude	− 22.07	− 69.57 to 25.43	0.35	− 45.05	− 90.95 to 0.84	**0.05**	− 39.54	− 86.32 to 7.23	0.09	− 76.73	− 130.05 to − 23.42	**0.005**	20.57	− 25.99 to 67.13	0.38
Model 1	− 20.15	− 68.21 to 27.89	0.40	− 44.99	− 90.82 to 0.84	**0.05**	− 37.41	− 84.37 to 9.54	0.11	− 78.54	− 131.84 to − 25.24	**0.004**	14.27	− 32.76 to 61.31	0.54
Model 2	− 29.28	− 77.9 to 19.33	0.23	− 43.44	− 89.02 to 2.13	0.06	− 37.66	− 84.55 to 9.23	0.11	− 83.61	− 136.91 to − 30.31	**0.002**	5.30	− 45.01 to 55.63	0.83
*Hb*															
Crude	0.67	0.10 to 1.24	**0.02**	0.02	− 0.53 to 0.58	0.92	**0.06**	− 0.49 to 0.63	0.81	0.53	− 0.12 to 1.19	0.11	0.47	− 0.07 to 1.02	**0.09**
Model 1	0.65	0.07 to 1.23	**0.02**	0.03	− 0.53 to 0.59	0.90	**0.09**	− 0.47 to 0.66	0.74	0.53	− 0.13 to 1.20	0.11	0.46	− 0.09 to 1.02	0.10
Model 2	0.61	0.02 to 1.19	**0.04**	0.03	− 0.52 to 0.59	0.90	**0.09**	− 0.47 to 0.67	0.73	0.56	− 0.11 to 1.23	0.10	0.41	− 0.18 to 1.01	0.17
*Postprandial BS*															
Crude	− 9.83	− 32.77 to 13.10	0.39	− 18.43	− 39.94 to 3.07	0.09	− 4.37	− 26.48 to 17.73	0.69	− 12.50	− 38.88 to 13.87	0.34	− 12.22	− 33.89 to 9.45	0.26
Model 1	− 6.52	− 29.15 to 16.11	0.56	− 19.03	− 39.95 to 1.89	0.07	− 4.43	− 26.06 to 17.19	0.68	− 13.46	− 39.18 to 12.25	0.30	− 14.93	− 36.18 to 6.31	0.16
Model 2	− 2.68	− 24.44 to 19.07	0.80	− 16.51	− 36.37 to 3.34	0.10	− 3.06	− 23.64 to 17.50	0.76	− 13.25	− 37.87 to 11.37	0.28	− 6.41	− 28.17 to 15.33	0.55
*Alb*															
Crude	− 2.48	− 7.63 to 2.69	0.34	− 0.14	− 5.03 to 4.74	0.95	− 3.28	− 8.20 to 1.63	0.18	− 7.04	− 12.82 to − 1.27	**0.01**	− 0.01	− 4.90 to 4.87	0.99
Model 1	− 1.93	− 7.12 to 3.26	0.46	− 0.20	− 5.08 to 4.67	0.93	− 3.26	− 8.18 to 1.66	0.19	− 7.75	− 13.31 to − 1.82	**0.01**	− 0.67	− 5.59 to 4.24	0.78
Model 2	− 2.38	− 7.50 to 2.82	0.37	0.24	− 4.55 to 5.04	0.92	− 3.08	− 7.90 to 1.74	0.20	− 7.16	− 12.80 to − 1.52	**0.01**	− 349	− 8.59 to 1.61	0.17
*SBP*															
Crude	− 4.97	− 12.38 to 2.44	0.18	− 9.09	− 15.94 to − 2.25	**0.01**	− .96	− 8.13 to 6.21	0.79	0.057	− 8.004 to 9.15	0.89	− 0.05	− 7.12 to 7.02	0.98
Model 1	− 4.59	− 11.94 to 2.57	0.21	− 9.22	− 15.95 to − 2.49	**0.008**	− 1.80	− 8.90 to 5.28	0.61	− 0.07	− 8.54 to 8.39	0.98	0.22	− 6.81 to 7.26	0.94
Model 2	− 5.72	− 13.231.78	0.13	− 9.87	− 16.62 to − 3.12	**0.005**	− 1.75	− 8.89 to 5.37	0.62	− 1.63	− 4.95 to 8.19	0.70	− 1.34	− 8.90 to 6.22	0.7
*DBP*															
Crude	− 6.50	− 11.91 to − 1.08	**0.01**	− 4.17	− 9.47 to 1.12	0.12	− 0.74	− 6.18 to 4.68	0.78	3.06	− 3.39 to 9.51	0.34	− 6.41	− 11.63 to − 1.20	**0.01**
Model 1	− 6.72	− 12.17 to − 1.26	**0.01**	− 4.14	− 9.44 to 1.14	0.12	− 1.008	− 6.42 to 4.44	0.71	2.92	− 3.50 to 9.39	0.37	− 6.25	− 11.51 to − 0.99	**0.02**
Model 2	− 7.40	− 12.97 to − 1.83	**0.01**	− 4.74	− 10.12 to 0.64	0.08	− 1.005	− 6.56 to 4.55	0.72	1.61	− 4.95 to 8.19	0.62	− 7.53	− 13.21 to − 1.86	**0.01**

Variables	Whole grain			Sodium			Red meat			Sweetened beverages		
	β	95% CI	*P* value	β	95% CI	*P* value	β	95% CI	*P* value	β	95% CI	*P* value
*FBS*												
Crude	4.81	− 16.04 to 25.67	0.64	18.21	− 6.60 to 43.03	0.14	13.79	− 34.13 to 6.54	0.18	2.89	− 22.72 to 16.93	0.77
Model 1	− 0.92	− 21.23 to 19.38	0.92	14.73	− 9.35 to 38.82	0.22	5.82	− 26.79 to 15.14	0.58	3.85	− 15.54 to 23.25	0.69
Model 2	3.26	− 16.94 to 23.47	0.74	16.79	− 8.83 to 42.43	0.19	1.02	− 22.82 to 20.77	0.92	3.41	− 15.80 to 22.63	0.72
*TG*												
Crude	− 15.94	− 42.86 to 10.97	0.24	2.85	− 35.37 to 29.67	0.86	− 2.33	− 29.08 to 24.41	0.86	7.82	− 17.88 to 33.52	0.54
Model 1	− 14.44	− 41.66 to 12.70	0.29	1.18	− 31.47 to 33.83	0.94	3.15	− 24.96 to 31.28	0.82	5.72	− 20.38 to 31.82	0.66
Model 2	− 13.17	− 41.77 to 15.42	0.36	1.20	− 33.58 to 35.99	0.94	5.11	− 25.99 to 36.22	0.74	4.93	− 22.37 to 32.24	0.72
*HDL*												
Crude	0.95	− 2.85 to 4.77	0.62	− 2.22	− 6.78 to 2.34	0.33	− 1.05	− 5.15 to 2.14	0.41	0.76	− 2.86 to 4.39	0.67
Model 1	0.73	− 3.18 to 4.65	0.71	− 2.63	− 7.29 to 2.02	0.26	− 2.08	− 5.99 to 1.82	0.29	1.11	− 2.63 to 4.86	0.55
Model 2	1.30	− 2.49 to 5.09	0.49	− 3.42	− 7.98 to 1.12	0.13	− 0.22	− 4.20 to 3.75	0.91	0.88	− 2.73 to 4.50	0.62
*LDL*												
Crude	− 4.24	− 17.32 to 8.83	0.52	9.76	− 25.38 to 5.58	0.21	2.26	− 10.55 to 15.08	0.72	2.06	− 14.51 to 10.38	0.74
Model 1	− 3.56	− 17.00 to 9.87	0.60	9.62	− 25.59 to 6.33	0.23	3.56	− 10.16 to 17.30	0.60	2.86	− 15.71 to 9.99	0.66
Model 2	− 5.64	− 18.98 to 7.69	0.40	13.76	− 29.96 to 2.44	**0.09**	0.57	− 13.65 to 14.80	0.93	− 0.48	− 13.21 to 12.25	0.94
*Total cholesterol*												
Crude	− 3.70	− 19.40 to 12.00	0.64	10.25	− 29.02 to 8.51	0.28	10.09	− 5.30 to 25.48	0.19	2.90	− 17.83 to 12.02	0.70
Model 1	− 3.93	− 20.11 to 12.24	0.63	9.60	− 28.85 to 9.65	0.32	12.82	− 3.61 to 29.27	0.12	3.14	− 18.61 to 12.32	0.68
Model 2	− 8.44	− 24.51 to 7.62	0.29	6.57	− 26.22 to 13.57	0.50	6.36	− 10.95 to 23.67	0.46	2.28	− 17.65 to 13.08	0.76
*HbA1C*												
Crude	0.36	− 0.21 to 0.93	0.21	0.11	− 0.58 to 0.81	0.73	− 0.45	− 1.01 to 0.10	0.10	− 0.14	− 0.69 to 0.41	0.61
Model 1	0.20	− 0.35 to 0.76	0.47	0.01	− 0.66 to 0.68	0.96	− 0.24	− 0.82 to 0.33	0.40	0.04	− 0.49 to 0.58	0.86
Model 2	0.20	− 0.37 to 0.78	0.49	0.05	− 0.66 to 0.76	0.88	− 0.22	− 0.84 to 0.40	0.48	− 0.01	− 0.57 to 0.53	0.94
*Creatinine*												
Crude	− 0.056	− 0.12 − 0.01	0.09	− 0.01	− 0.09 to 0.06	0.64	0.05	− 0.11 to 0.01	0.12	− 0.02	− 0.09 to 0.03	0.36
Model 1	− 0.06	− 0.13 − 0.006	0.07	− 0.02	− 0.10 to 0.05	0.55	0.05	− 0.12 to 0.02	0.16	− 0.02	− 0.09 to 0.04	0.45
Model 2	− 0.07	− 0.14 − 0.01	**0.02**	− 0.01	− 0.09 to 0.07	0.81	0.096	− 0.16 to 0.02	**0.009**	− 0.02	− 0.08 to 0.04	0.51
*BUN*												
Crude	0.73	− 1.13 to 2.60	0.43	− 1.79	− 4.02 to 0.42	0.11	0.99	− 0.85 to 2.84	0.29	0.14	− 1.63 to 1.92	0.87
Model 1	0.66	− 1.27 to 2.58	0.49	− 1.85	− 4.13 to 0.42	0.10	1.41	− 0.56 to 3.38	0.16	0.26	− 1.58 to 2.11	0.77
Model 2	0.16	− 1.74 to 2.06	0.86	− 1.51	− 3.80 to 0.76	0.19	0.63	− 1.41 to 2.68	0.53	0.26	− 1.53 to 2.07	0.76
*ACR*												
Crude	51.91	5.95 to 97.86	0.30	15.66	− 72.07 to 40.57	0.58	− 16.41	− 62.41 to 29.59	0.48	21.57	− 22.94 to 66.08	0.33
Model 1	46.38	− 0.34 to 93.11	0.20	− 19.58	− 76.36 to 37.18	0.49	− 12.14	− 60.83 to 36.54	0.62	29.97	− 15.17 to 75.11	0.19
Model 2	38.55	− 9.22 to 86.32	0.11	− 6.08	− 64.52 to 52.53	0.83	− 38.04	− 89.28 to 13.19	0.14	29.93	− 15.72 to 75.58	0.19
*Hb*												
Crude	0.44	− 0.11 to 1.002	0.11	− 0.05	− 0.73 to 0.62	0.87	0.96	0.44 to 1.49	0.40	− 0.18	− 0.17 to 0.35	0.49
Model 1	0.48	− 0.08 to 1.54	0.09	− 0.07	− 0.75 to 0.61	0.83	0.97	0.41 to 1.53	0.58	− 0.21	− 0.76 to 0.33	0.44
Model 2	0.40	− 0.17 to 0.98	0.16	0.002	− 0.70 to 0.71	0.99	0.91	0.31 to 1.52	0.60	− 0.20	− 0.76 to 0.34	0.46
*Postprandial BS*												
Crude	14.85	− 6.90 to 36.62	0.17	17.66	− 8.47 to 43.79	0.18	19.07	− 40.48 to 2.34	0.08	3.03	− 23.85 to 17.85	0.77
Model 1	10.35	− 11.24 to 31.94	0.34	15.86	− 9.85 to 41.58	0.22	11.46	− 33.82 to 10.88	0.31	2.41	− 18.31 to 23.14	0.81
Model 2	16.23	− 4.46 to 36.93	0.12	16.21	− 10.63 to 43.06	0.23	1.50	− 24.25 to 21.24	0.89	0.34	− 20.28 to 19.60	0.97
*Alb*												
Crude	− 6.85	2.11 to 11.59	**0.005**	− 1.66	− 7.57 to 4.23	0.57	− 0.32	− 5.21 to 4.57	0.89	− 2.18	− 6.84 to 2.47	0.35
Model 1	− 6.22	1.40 to 11.04	**0.01**	− 2.49	− 8.42 to 3.42	0.40	− 0.39	− 4.74 to 5.54	0.87	− 1.29	− 6.4 to 3.45	0.59
Model 2	− 5.66	0.84 to 10.48	**0.02**	− 2.56	− 8.56 to 3.43	0.39	− 1.73	− 7.10 to 3.63	0.52	− 0.70	− 5.41 to 4.01	0.76
*SBP*												
Crude	3.74	− 3.34 to 10.82	0.29	3.01	− 11.54 to 5.51	0.48	2.13	− 4.93 to 9.19	0.55	0.88	− 5.88 to 7.64	0.79
Model 1	3.62	− 3.45 to 10.70	0.31	3.93	− 12.40 to 4.53	0.35	1.29	− 6.05 to 8.65	0.72	1.27	− 5.52 to 8.07	0.71
Model 2	3.02	− 4.23 to 10.27	0.41	3.39	− 12.18 to 5.39	0.44	1.34	− 9.24 to 6.55	0.73	1.55	− 5.36 to 8.47	0.65
*DBP*												
Crude	− 6.47	− 11.72 to − 1.22	**0.01**	6.58	− 13.19 to − 0.51	**0.03**	0.78	− 6.07 to 4.50	0.76	2.62	− 7.72 to 2.48	0.31
Model 1	− 6.21	− 11.53 to − 0.88	**0.02**	7.10	− 13.48 to − 0.72	**0.02**	2.23	− 7.80 to 3.34	0.42	3.20	− 8.39 to 1.98	0.22
Model 2	− 6.92	− 12.40 to − 1.44	**0.01**	6.81	− 13.52 to − 0.10	**0.04**	4.07	− 10.10 to 1.95	0.18	3.42	− 8.76 to 1.92	0.20

Obtained from General Linear Model (GLM). Significant items with a* P* value ≤ 0.05 are bolded

Model 1: Adjusted for energy intake, age, physical activity, body mass index

Model 2: model 1 + diabetes duration, cardiovascular diseases history, and drug usage (angiotensin receptor blockers; angiotensin converting enzyme inhibitors, beta-blockers, metformin, sulphonyl urea, and insulin)

*CI* Confidence interval, *Cr* Creatinine, *DASH* Dietary approaches to stop hypertension diet, *DBP* Diastolic blood pressure, *SBP* Systolic blood pressure, *FBS* Fasting blood sugar, *HbA1c* Glycosylated hemoglobin, *HDL* High-density lipoprotein, *LDL* Low-density lipoprotein, *TC* Total cholesterol, *TG* Triglycerides, *Alb* Albumin, *BS* Blood sugar, *ACR* Albumin creatinine ratio, *BUN* Blood urea nitrogen

## Discussion

The present study demonstrated that higher adherence to the DASH diet yielded a 74% reduction in the odds of DN. These findings support our hypothesis that people who adhere to the DASH diet decrease the risk of DN among the adult population. After adjusting for confounders, such as energy intake, age, physical activity, and BMI, the DASH diet remained negatively associated with the odds of DN. Thus, the DASH diet appears to be effective in controlling the risk of DN among adults. To our knowledge, the present study is the first to examine the effects of the DASH eating pattern and subcategories on DN and other biochemical markers among type 2 diabetes.

As the incidence of T2D increases, efforts to stop the progression of diabetes to diabetic kidney disease and ESRD are essential [28]. There are several main risk factors for DN, including, family history, ethnicity, gestational diabetes, dyslipidemia, hyperglycemia, obesity, being a smoker, high blood cholesterol, hypertension, and insulin resistance. The DASH diet, which promotes the consumption of fruits, vegetables, low-fat dairy products, and sodium restriction, was created for people with hypertension [29]. This diet is rich in antioxidants, unsaturated fatty acids, fiber, and low-fat dairy, all of which may be crucial for reducing insulin resistance, inflammatory levels, and metabolic disruption [30].

In the present study we found that, there was an inverse relationship between DASH score and serum Cr. In line with our study, a cross-sectional study of participants with T2D in Taiwan demonstrated that greater adherence to a healthy diet and higher intake of fish and vegetables was negatively correlated with serum Cr, and positively associated with eGFR [31]. Given our results, nuts and legumes were inversely associated with ACR and Alb. The DASH diet plan’s positive benefits on metabolic parameters could possibly be attributed to a larger consumption of legumes. Additionally, the DASH diet includes more soy products, which may be linked to better cardio-metabolic health and lower plasma levels of CRP [32]. The DASH study shown that despite an increase in calories from protein, the DASH diet did not raise urinary albumin excretion rate [33].

In our study, we indicated an inverse association between DASH score and DBP. Previous investigations indicated that this diet can be used to control hypertension and blood glucose, according to recent studies [11, 12]. It appears that greater adherence to plant-based diets and less consumption of processed foods can have a positive impact on DN [13]. In the DASH diet, individuals are encouraged to consume whole grains, fruits, vegetables, low-fat dairy products, legumes, seeds, fish, and poultry (lean meats), whilst consumption of saturated fat, red meats, sweets, and SSBs is restricted [14, 15]. Higher amounts of protein, fiber, magnesium, calcium, potassium, antioxidant components, and unsaturated fatty acids in this diet, can help to reduce the risk of diabetes [34]. Several studies have demonstrated the association between the DASH diet and the risk of diseases; for instance, the insulin resistance atherosclerosis study demonstrated that adherence to the DASH dietary pattern may have the potential to prevent T2D [11].

In the present study, vegetables maintained negative association with ACR and SBP after adjusting for confounding variables. Additionally, in an adjusted model, Fruits also displayed a negative association with Cr.

A prospective cohort study among south Korean found that intake of a diet rich in fruits, and vegetables were associated with a decreased risk of CKD [35]. The DASH trial demonstrated that dietary patterns rich in vegetables, fruit, and low-fat dairy products can reduce systolic and diastolic blood pressure [36]. Hypertension is a risk factor for kidney diseases and also DN, which is reduced by adherence to the DASH diet, according to recent studies [21]. Concomitantly intervention studies have revealed that the DASH diet has beneficial effects on total and LDL cholesterol, insulin sensitivity, and weight management [37, 38]. Moreover, in postmenopausal women without diabetes, better adherence to the DASH diet was associated with a lower prevalence of metabolic syndrome, according to a cross-sectional study using Korean National Health and Nutrition Examination Survey (KNHANES) [39]. Additionally, the DASH diet can significantly protect against cardiovascular disease (CVD) 20%, Coronary heart disease (CHD) 21%, stroke 19%, and Heart failure (HF) 29%, according to a systematic review and meta-analysis of observational prospective studies [38]. In addition, elderly adults with a high intake of the DASH-style diet were reported to have lower odds of having Chronic kidney disease (CKD), according to a cross-sectional study using the KNHANES data [40]. Furthermore, the DASH diet has been associated with a lower risk of kidney damage, particularly the decline in glomerular filtration rate [35, 41]. Additionally, the relationship between the DASH diet and CKD progression or CKD-related complications has not been reported in East Asia [42]. Some clinical trial studies have demonstrated inconsistent results regarding the relationship between the DASH diet and diabetes [43, 44]. In addition, adherence to the DASH diet with a routine exercise program can affect diabetes in persons with hypertension and overweight [38]. The National Health and Nutrition Survey, Japan, found that better adherence to the DASH diet was inversely associated with metabolic risk factors, including waist circumference, TC, LDL cholesterol, and BMI [45]. Generally, the potential direct relationship between greater adherence to the DASH and kidney function has been largely explained by positive impacts of this diets on levels of cardiometabolic risk factors including blood pressure [46, 47], glycemic control [11], and lipid profile [48].

Furthermore, concordant with our hypothesis, there is an association between the DASH diet and the risk of DN. Evidence regarding the association between diets and DN patients is scarce; however, a case–control study among 105 women demonstrated that adherence to the DASH was inversely and dose-dependently associated with risk of DN [49]. Moreover, a prospective cohort study revealed that a DASH-style diet was associated with a lower risk for CKD and DN [50]. In addition, a prospective study using data from the Singapore Chinese Health Study found that greater adherence to a DASH-style diet was associated with a 29% lower risk of developing T2D [51]. The DASH dietary pattern may be beneficial both in the prevention and management of diabetes mellitus nephropathy [41].

The strengths of our study include that, to best our knowledge, this is the first study to have examined the association of DASH diet and risk of DN among Iranian adults, as well as considering a wide range of confounders. On the other hand, our study has several limitations that should be noted. The case–control design of the study precludes casual inferences. The second limitation is the use of FFQ for dietary assessment, which might result in misclassification of participants. Although we controlled for several confounding variables, the existence of residual confounding cannot be excluded. Furthermore, as in all epidemiologic studies, random errors might affect our results because diet and lifestyle information might be collected with some degree of error.

## Conclusion

We found that DASH diet adherence may be associated with lower odds of DN. Further studies, with large sample sizes, are needed to confirm the veracity of this association.

## Data Availability

The authors confirm that the data supporting the findings of this study are available within the manuscript and in the included tables.

## Abbreviations

ACR: Albumin-to-creatinine ratio
ADA: American Diabetes Association
ARBs: Angiotensin receptor blockers
ACEIs: Angiotensin-converting enzyme
ANCOVA: Analysis of covariance
BMI: Body mass index
BUN: Blood urea nitrogen
BP: Blood pressure
CI: Confidence interval
CKD: Chronic kidney disease
Cr: Creatinine
CVD: Cardiovascular disease
CHD: Coronary heart disease
DASH: Dietary approaches to stop hypertension diet
DBP: Diastolic blood pressure
DN: Diabetic nephropathy
ESRD: End-stage renal disease
FBG: Fasting blood glucose
FBS: Fasting blood sugar
FFQ: Fod-frequency questionnaire
2hrBG: 2-Hour post-load blood glucose
HF: Heart failure
HbA1c: Glycosylated hemoglobin
HDL: High-density lipoprotein
KNHANES: Korean National Health and Nutrition Examination Survey
LDL: Low-density lipoprotein
IPAQ: International Physical Activity Questionnaire
OR: Odds ratio
PA: Physical activity
SSBs: Sugar-sweetened beverages
SDs: Standard deviations
T2D: Type 2 diabetes
TC: Total cholesterol
TG: Triglycerides
TUMS: Tehran University of Medical Sciences
